# Mg-enriched nutrient management enhances phyllosphere bacterial diversity, community structure, and functional traits in pomelo orchards

**DOI:** 10.1016/j.crmicr.2025.100476

**Published:** 2025-09-25

**Authors:** Muhammad Atif Muneer, Rong Huang, Yan Xiaojun, Ziqin Pang, Muhammad Zeeshan Munir, Baoming Ji, Liangquan Wu, Chaoyuan Zheng

**Affiliations:** a International Magnesium Institute, College of Resources and Environment, Fujian Agriculture and Forestry University, Fuzhou, Fujian 350002, China; b Institute of Bast Fiber Crops, Chinese Academy of Agricultural Sciences, Changsha 410221, China; c State Key Laboratory of Nutrient Use and Management, College of Resources and Environmental Sciences, Key Laboratory of Plant- Soil Interactions, Ministry of Education, China Agricultural University, Beijing 100193, China; d Xianghu Laboratory, Hangzhou 311231,China; e School of Environment and Energy, Peking University Shenzhen Graduate School, 2199, Lishui Rd, Shenzhen 518055, China; f College of Grassland Science, Beijing Forestry University, Beijing, China

**Keywords:** Endophytes, Phyllosphere, Bacteria, Magnesium, Nutrient management, Pomelo

## Abstract

Phyllosphere, the aerial surface of plants, harbors a tremendous diversity of microbes that significantly influences plant health and ecosystem functionality. Nevertheless, a holistic understanding of the effects of nutrient management, particularly magnesium (Mg) supplementation, on phyllosphere endophytes remains elusive. Herein, we conducted a five-year field experiment to investigate the impact of different nutrient management practices, including farmer practice of high N.P.K input (FP), reduced N.P.K input (OPT), and OPT supplemented with Mg (OPT+Mg), on the phyllosphere endophytic bacterial community (Illumina 16S sequencing) associated with fruit and leaf of pomelo trees across different growth stages, i.e., June (S1), July (S2), and September (S3). The results demonstrated that Mg-enriched nutrient management (OPT+Mg), significantly enhanced microbial diversity and restructured community composition, with notable increases in the relative abundance of beneficial phyla such as Actinobacteriota, Bacteroidota, and Chloroflexi compared with FP across all growing seasons. Similarly, both OPT and OPT+Mg generally supported higher microbial diversity than FP in fruit and leaf samples. Principal Coordinate Analysis (PCoA) and PERMANOVA confirmed significant differences in bacterial community structures among treatments. Ternary plot analysis further indicated the enrichment of beneficial genera under OPT and OPT+Mg treatments, which can play key roles in promoting plant growth and enhancing resilience against various stresses. In addition, Mg application was associated with more complex co-occurrence networks, highlighting its impact on promoting robust microbial communities in leaf and fruit samples. Functional predictions (FAPROTAX) suggested that Mg application was associated with higher predicted carbon and nitrogen cycling functions and lower predicted relative abundances of potential plant pathogens and intracellular parasites. This study is the first to provide long-term field-based evidence of how Mg supplementation modulates phyllosphere microbiota in fruit orchards, offering critical insights into microbiome-informed nutrient strategies. These findings highlight the unexplored role of Mg in shaping aerial plant microbiomes and present a novel avenue for enhancing sustainable pomelo production through targeted nutrient-microbiome interventions.

## Highlights

• Mg reshapes pomelo phyllosphere microbiota over different growth stages
• Mg increases FAPROTAX-predicted beneficial taxa and lowers pathogen taxa
• Mg improves predicted microbial functions related to carbon and nitrogen cycling
• Mg-enriched inputs could pave the way for microbiome-driven pomelo sustainability

## Graphical abstract

**Figure:**
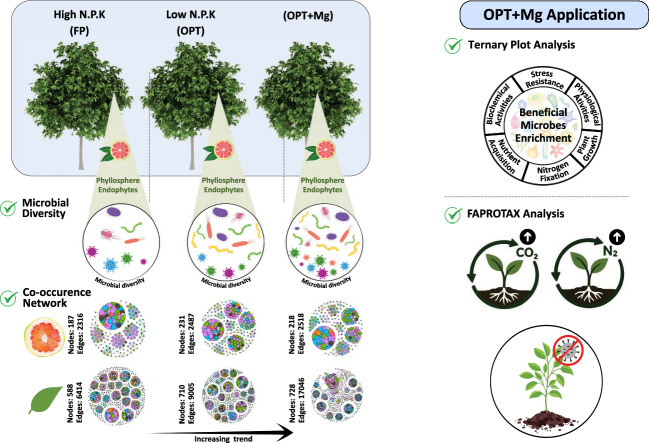

## 1 Introduction

Plant-associated microbiomes play a pivotal role in enhancing crop productivity, nutrient acquisition, and resilience to biotic and abiotic stresses, thereby contributing to the sustainability of modern agricultural systems (Kumar et al., 2023; Nadarajah and Abdul Rahman, 2023). These microbial communities colonize different plant compartments, forming spatially distinct microbiomes in the phyllosphere, rhizosphere, and endosphere (Dastogeer et al., 2020). Among these, the phyllosphere microbiome, inhabiting the aerial parts of plants such as fruits, leaves, flowers, and seeds, represents a vast yet relatively unexplored ecological niche (Koskella, 2020). The phyllosphere microbiome includes both epiphytic microbes, living on plant surfaces, and endophytic microbes, residing within plant tissues. The phyllosphere provides a unique interface between plants and the atmosphere, where microbial activities influence physiological functions, disease resistance, and even nutrient cycling (Bringel and Couée, 2015).

Phyllosphere endophytes, which inhabit internal leaf tissues without causing harm, play crucial roles in promoting plant growth and stress tolerance. Numerous studies have revealed that phyllosphere endophytes positively affect various aspects of plant fitness, including leaf functions and longevity, seed mass, apical growth, flower onset, and fruit maturity (Zhan et al., 2022; Zhu et al., 2022). Consequently, these endophytes substantially improve crop yield and quality, making them pivotal for agricultural sustainability. However, the assembly and dynamics of phyllosphere communities are highly sensitive to both environmental conditions and anthropogenic factors, including land-use change, urbanization, and agricultural intensification (Liu et al., 2025; Zhu et al., 2022). While substantial research has examined the effects of climate, geography, and host genotype on these communities, the influence of nutrient management practices, particularly fertilization, remains insufficiently explored.

In modern agriculture, chemical fertilizers, including nitrogen (N), phosphorus (P), and potassium (K) fertilizers, have been widely used in crop production to improve the yields (Yan et al., 2023). Global agricultural production is projected to increase by 70 % to feed the growing population (Haskett et al., 2021; Singh et al., 2020). This growth likely means that the use of chemical fertilizers in agricultural production will double in the near future. However, such intensive fertilization may not only lead to soil degradation, such as acidification, and environmental pollution (Raza et al., 2020), but also negatively impact plant-associated microbial communities, including shifts in phyllosphere microbial diversity, community structure, and functional traits (Thapa and Prasanna, 2018). Moreover, over-fertilization can impair aboveground microbial communities by altering the plant’s nutrient status, leaf chemistry, and immune responses, ultimately affecting the phyllosphere microbiome (Zhu et al., 2022). Therefore, optimizing nutrient management is essential not only for soil and environmental health but also for maintaining the integrity and function of phyllosphere microbial communities, contributing to sustainable agricultural practices.

Magnesium (Mg), an often overlooked secondary macronutrient in agricultural systems, plays an essential role in plant growth, development, and quality formation (Li et al., 2023a). Mg is an essential part of chlorophyll and is critical for photosynthesis, protein synthesis, enzymatic activation, and carbohydrate transport (Cakmak, 2013). Despite its importance, Mg deficiency is widespread, especially in acidic and sandy soils where leaching is prevalent (Cakmak and Yazici, 2010; Gerendás and Führs, 2013). Mg deficiency is more common in soils where only N.P.K fertilizer application is adopted, and as a result, it hinders the absorption of essential nutrients by crop plants (Chen et al., 2022; Farhat et al., 2016). However, the use of Mg fertilizer in agriculture has increased in the last decade due to a growing understanding of its importance. For instance, the positive impact of Mg-based fertilizers on the alleviation of abiotic stressors, such as photooxidative damage and Al toxicity, as well as heat stress, has long been documented in the literature (Cakmak and Kirkby, 2008; Mengutay et al., 2013; Muneer et al., 2024). In addition, Mg fertilizer has been found to increase the yield and quality of various crop species such as soybean, potato, sugar beet, cabbage, oil crops, lentils, and tea (Azizi et al., 2011; Deliboran et al., 2011; Zhang et al., 2022). Importantly, Mg may also shape the phyllosphere microbiome indirectly. By boosting photosynthesis and chlorophyll formation, Mg changes the production and composition of leaf exudates (e.g., sugars, organic acids), which in turn influences the resources available for microbes living on plant surfaces and inside leaves (Cakmak, 2013; Guo et al., 2010). However, few studies have explicitly focused on how Mg fertilization could affect phyllosphere microbial diversity, community composition, and functioning.

It is well documented that N fertilizers significantly impact inter‑kingdom interactions between bacteria and fungi in the rhizosphere (Li et al., 2021; Zhao et al., 2019). Similarly, phyllosphere communities, as a vital component of the plant microbiome, are highly responsive to changes in soil fertility (Trivedi et al., 2020; Xiang et al., 2020). It has been documented that excessive use of N fertilizer leads to an increasing incidence of plant diseases (Lekberg et al., 2021; Walters and Bingham, 2007), and N fertilization is associated with the enrichment of plant pathogens in the leaf endosphere (Xiong et al., 2021). Though these studies provide insight into N fertilization, the regulatory roles of Mg-based fertilization on the phyllosphere bacterial community are largely still unknown, representing a substantial research gap in our understanding of nutrient management and its impact on plant-microbe interactions.

Pinghe County is in Southeast China and is well known for its pomelo (*Citrus grandis*) production, the third most important citrus variety after *Citrus reticulata* and *Citrus sinensis* (Zhang et al., 2021). Pomelo production is a major source of income and has long been an integral part of the livelihoods of residents in this area (Li et al., 2015). However, the economic benefits of pomelo cultivation in recent years have led to large annual applications of N.P.K fertilizers to enhance crop yields, often at the expense of essential macronutrients such as Mg (Cakmak and Yazici, 2010). Therefore, this overuse and unreasonable fertilizer application result in various soil problems, such as low soil pH and reduced soil Mg availability (Guo et al., 2010, 2019) Hence, Mg fertilizer should be an integral component of soil management practices. Although Mg fertilizer has excellent potential in increasing pomelo productivity and quality (Zhang et al., 2021), its impact on the phyllosphere bacterial diversity and community composition of pomelo orchards has not yet been investigated.

The present study was conducted to bridge this knowledge gap by assessing the phyllosphere endophytic bacterial diversity, community composition, and functions associated with leaf and fruit samples of pomelo trees from a five-year field experiment under various nutrient management practices. Bacterial communities were assessed by 16S ribosomal RNA (rRNA) sequencing. The overall objectives of this study were to: 1) evaluate the effects of different nutrient management practices on phyllosphere bacterial diversity, composition, and network patterns; 2) identify the core phyllosphere bacterial community; 3) examine the functioning of the phyllosphere bacterial community under different management practices across different growth stages.

## 2 Materials and methods

### 2.1 Experimental design and sampling

The field experiment was carried out in Pinghe County (24°02′-24°35′N, 116°54′-117°31′E), in Fujian Province, China. The climate in the study region is characterized by a subtropical oceanic monsoon climate, with an average annual rainfall of 1600 to 2000 mm and an average annual temperature of 17.5 °C to 21.3 °C. The primary soil types found in this region are acidic red soils, categorized as Ferralsols or Oxisols according to the FAO and USDA classification systems, respectively.

**Fig. 1:**
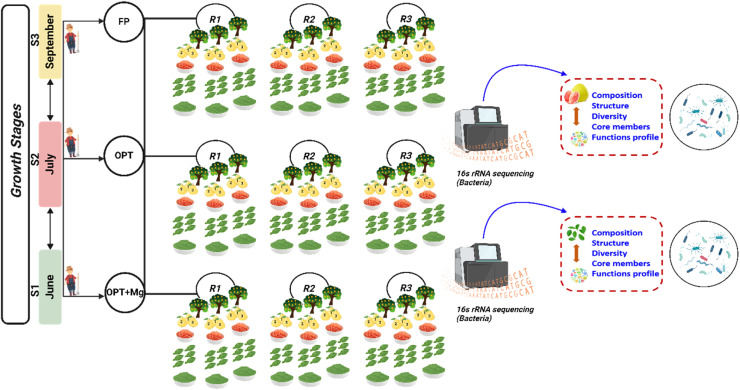
Schematic diagram of the experiment. Here S1, S2, S3 represent various growth stages in June, July, September, respectively. While, FP, OPT, and OPT+Mg represents different treatments.

This long-term fertilization experiment, initiated in 2019 and samples were collected during the 2023 growing season (representing the fifth year of continuous treatment application), used a randomized block design with the following three treatments: (1) Farmer practice (FP), a high-input reference representing local practice with high N.P.K fertilizer and organic matter inputs; (2) Optimized (OPT) treatment with reduced/balanced N.P.K fertilizer input without organic matter; (3) OPT+Mg, which is identical to OPT but supplemented with Mg fertilizer (Table 1). Three replications were set for each treatment, and for each replication, three healthy pomelo trees were selected to collect fruit and leaf samples. For fruit samples, three pomelo fruits were selected from each tree, and the pulp from each fruit was obtained and mixed to make one composite sample. For leaf, six leaves were selected from each tree and mixed to make a leaf composite sample (Fig. 1). All leaf samples were stored with ice packs immediately after collection, and transported to the laboratory within 12 h, and stored at −80 °C for further processing. All samples were surface-sterilized prior to DNA extraction to target endophytic bacteria. Specifically, fruit and leaf tissues were rinsed in sterile water, followed by immersion in 70 % ethanol, then 1 % sodium hypochlorite, and rinsed three times in sterile water. Samples were collected during the different growth seasons in 2023, i.e., June (S1), July (S2), and September (S3). The planting density at the experimental site was 1218 trees per hectare, and the plant material was 14-year-old ‘Guanximiyou’ pomelo trees grafted onto ‘Sour pomelo (*Citrus grandis*) rootstocks.

**Table 1:** Detail of different treatments.

Treatments	N	P_2_O_5_	K_2_O	MgO	Organic matter
	kg.hm^−2^	kg.hm^−2^	kg.hm^−2^	kg.hm^−2^	kg.hm^−2^
FP	1084	914	906	0	7500
OPT	230	0	230	0	0
OPT+Mg	230	0	230	67.5	0

### 2.2 DNA extraction, PCR amplification, illumina miseq sequencing and data processing

Genomic DNA was extracted from leaf and fruit samples using a DNA extraction kit (MoBio, Carlsbad, USA). DNA samples were quantified with a NanoDrop 2000 spectrophotometer (Thermo Scientific, Waltham, MA, USA), and the quality of extracted DNA was evaluated by electrophoresis on 1 % agarose gel and purified using the AxyPrepDNA Gel Extraction Kit (Axygen Biosciences, USA). The 16S rRNA gene fragment was amplified using the primers: 799F (5-AACMGGATTAGATACCCKG-3) and 1193R (5−ACGTCATCCCCACCTTCC−3). This study utilized the 799F_1193R primer pair to amplify a target region of approximately 394 bp. The 799F–1193R primer set was selected because it minimizes amplification of plant-derived chloroplast and mitochondrial 16S rRNA sequences, thereby enriching for bacterial reads and improving resolution of plant-associated bacterial communities (Bodenhausen et al., 2013; Chelius and Triplett, 2001). The PCR conditions were as follows: 94 °C for 3 min for initial denaturation, followed by 30 cycles at 94 °C for 40 s, 56 °C for 60 s, and 72 °C for 10 min for the final extension. Subsequently, each sample was sequenced on an Illumina MiSeq platform (Majorbio, Shanghai, China), and the paired-end reads were merged using FLASH software based on the sample-specific barcodes. For classification and annotation, all sequences were grouped into operational taxonomic units (OTUs) based on 97 % similarity. For each OTU, representative sequences were selected and annotated using the ribosomal digital library (Wang et al., 2007). During processing, low‑quality sequences were removed; only sequences longer than 200 nucleotides, with accurate base calls and an average quality score ≥ Q 20, were retained. Sequencing was performed with PE300 sequencing platform, resulting in an average read length of 375 bp. Across 54 samples, a total of 4490,889 sequences were generated, yielding over 1.68 billion bases. The sequencing depth per sample varied, with individual sample sequence counts ranging from approximately 45,000 to over 126,000. The average sequence length across all samples was consistent at around 375 bp. Finally, all sequences were clustered using 97 % nucleotide similarity. The bacterial sequences were classified and annotated using the SILVA digital library (SILVA Release 138). The sequencing data is available at NCBI BioProject SRA database under the accession number PRJNA1147855.

### 2.3 Statistical analyses

For downstream statistical analyses, QIIME (Quantitative Insight into Microbial Ecology) and R software (version 4.0.3) were employed to investigate the richness (ACE) and diversity (Shannon index) of the endophytic bacterial communities. To check the significant differences in alpha diversity indices among different treatments, the Kruskal–Wallis (KW) rank-sum test was employed. Beta diversity was assessed by the PCoA (principal coordinate analysis) based on Bray-Curtis dissimilarity to evaluate the differences in microbial community composition among different treatments. Moreover, PERMANOVA (Permutational analysis of variance) was used to evaluate the significant differences in bacterial communities among treatments and growth seasons. Ternary plot analysis was conducted using the R language-based packages ggtern and grid, an extension of the packageggplot2, to explore enriched bacterial communities under the different fertilizer treatments. Co-occurrence networks were constructed based on Spearman’s correlation; OTUs with significant correlations were chosen. These correlations were revealed by pairwise analysis of taxa abundance and resulted in a highly complex network thereby each node depicted the phylum, whereas the inter-node (stand among the nodes) represented the significant correlation between the nodes. For visualization and modularity of co-occurrence, Gephi V0.9.2 was used. Nodes with a high degree and relative abundance were categorized as keystone species in the co-occurrence network (Zhang et al., 2020). To predict the potential functions of the phyllosphere bacterial community, FAPROTAX (functional annotation of prokaryotic taxa) was used with default settings (Louca et al., 2016). Because FAPROTAX provides taxonomy-based predictions of functional potential rather than direct measurements of gene expression or process rates, the results were interpreted cautiously as indicative of potential functions rather than measured activities.

## 3 Results

### 3.1 Relative abundance of phyllosphere bacterial community

**Fig. 2:**
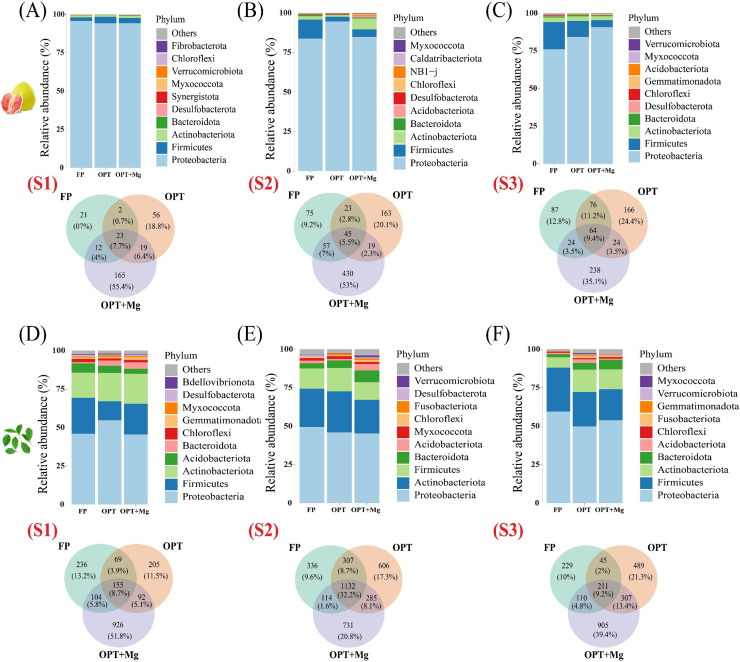
Relative abundance and richness of phyllosphere bacterial communities under different treatments across various growth stages; (A-C) fruit sample; (D-F) leaf sample. Here S1, S2, S3 represent various growth stages in June, July, September, respectively. While, FP, OPT, and OPT+Mg represents different treatments.

The relative abundance of phyllosphere bacterial community in fruit and leaf samples across different growth stages (S1, S2, S3) was analyzed under different treatments (Fig. 2). For fruir samples, Proteobacteria remained dominant under all treatments with minor variations. During the first growth stage, OPT substantially increased the relative abundance of Firmicutes (4.28 %) compared with FP (2.27 %), representing a shift in the phylloshere bacterial community structure. OPT+Mg further enhanced this shift, maintaing a higher level of Firmicutes (3.48 %) compared with FP (Fig. 2A). In the second growth stage, OPT exhibited a significant increase in relative abundance of Proteobacteria (94.60 %) compared to FP (83.73 %). This trend was slightly reversed under the OPT+Mg (84.76 %) but still maintained a higher abundance than FP. Interestingly, Actinobacteriota had a higher relative abundance under the OPT+Mg (6.98 %) compared to FP (2.12 %) and OPT (1.29 %), signifying the positive impact of Mg supplementation (Fig. 2B). The patterns observed in the first two growth stages were consistent with the third growth stage. For instance, Proteobacteria remained dominant under all treatments, and Firmicutes increased slightly under the OPT (4.28 %) and OPT+Mg (3.48 %) compared to FP (2.27 %). The relative abundance of Actinobacteriota also increased under OPT+Mg (1.37 %) compared to FP (1.14 %) (Fig. 2C).

For leaf samples, during the first growing season (S1), Proteobacteria was the predominant phylum across all treatments. The relative abundance of Proteobacteria was highest in the OPT treatment (54.66 %) compared to FP (46.01 %) and OPT+Mg (45.52 %). Notably, the OPT+Mg treatment showed a substantial presence of Actinobacteriota (19.51 %), Firmicutes (19.88 %), and Bacteroidota (4.14 %), which were higher than FP (Fig. 2D). In the second growing season (S2), Proteobacteria remained the most abundant phylum, with relative abundances of 49.48 % in FP, 45.94 % in OPT, and 45.26 % in OPT+Mg. The OPT+Mg treatment exhibited a higher relative abundance of Bacteroidota (7.65 %) compared to OPT (4.77 %) and FP (3.33 %). Additionally, Acidobacteriota was significantly more abundant in the OPT+Mg treatment (4.14 %) than in OPT (1.03 %) and FP (1.68 %) (Fig. 2E). In the third growing season (S3), Proteobacteria dominated in all treatments. The OPT+Mg treatment again showed a notable increase in the relative abundance of Bacteroidota (6.10 %) and Chloroflexi (1.18 %) compared to FP and OPT (Fig. 2F). These results suggest that the OPT and OPT+Mg treatments support a more diverse and potentially beneficial microbial community, which could enhance plant health and growth. The increased relative abundances of Actinobacteriota, Bacteroidota, and Chloroflexi under OPT and especially under OPT+Mg treatment indicate the positive effect of Mg addition in optimizing the microbial environment, promoting nutrient cycling, disease suppression, and plant growth. Therefore, incorporating magnesium into optimized treatment regimes fosters a more robust microbial community, improving pomelo plant performance across different growing seasons. Moreover, in each season for both fruit and leaf samples, the OPT+Mg consistently harbors the highest number of unique OTUs, indicating a substantial increase in microbial diversity with Mg supplementation. These results highlight that Mg significantly alters the microbial community structure and enhances microbial diversity across different growing seasons, with each season showing specific community compositions and responses to the treatments.

### 3.2 Changes in phyllosphere bacterial richness and diversity

**Fig. 3:**
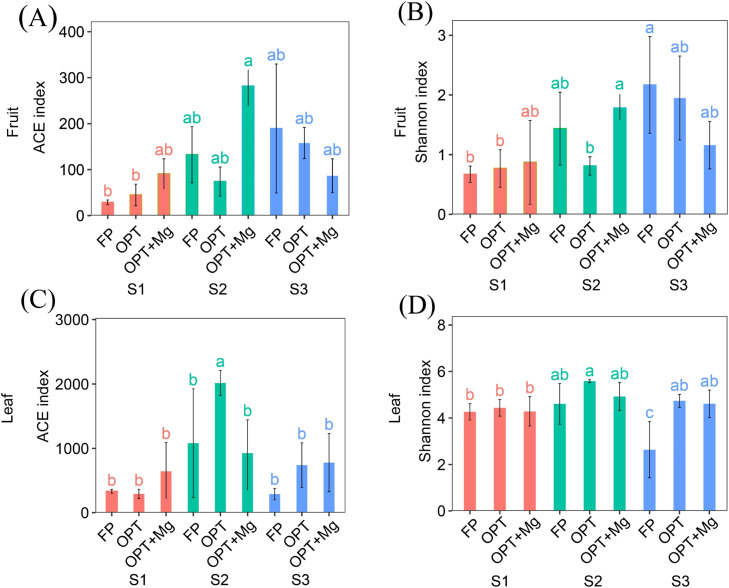
The alpha diversity indices under different treatments across various growth stages for leaf (A) ACE index; (B) Shannon index; and fruit (C) ACE index; (D) Shannon index. Here S1, S2, S3 represent various growth stages in June, July, September, respectively. While, FP, OPT, and OPT+Mg represents different treatments.

This study aimed to evaluate the influence of different optimized management practices on bacterial alpha diversity across three growth seasons by employing the ACE and Shannon diversity indices in both fruit and leaf samples (Fig. 3). The ACE index, which quantifies species richness, and the Shannon index, which quantifies species diversity, exhibited distinct patterns among different treatments and growing seasons. In fruit samples, both indices were lowest at S1 across all treatments and increased sharply at S2 (Fig. 3A, B). OPT+Mg consistently showed the highest richness and diversity, indicating a positive effect of Mg supplementation in enhancing community diversity. By S3, diversity declined slightly relative to S2 but remained higher than S1. Although FP had the numerically highest Shannon index at S3, statistical grouping indicated no significant differences among FP, OPT, and OPT+Mg. This suggests that the fruit development stage strongly influences microbial diversity, with optimized management, particularly OPT+Mg, having the clearest impact during the S2 stage. Overall, higher microbial diversity was observed in the optimized treatments, especially with Mg addition, suggesting that improved nutrient management practices enhance microbial diversity in fruit samples.

Regarding leaf samples, during the initial growth phase (S1), applying OPT +Mg led to a considerable increase in the ACE index (Fig. 3C) relative to both the FP and OPT. Consequently, this suggests an elevated level of species richness resulting from incorporating Mg. During the second period of growth (S2), the OPT treatment exhibited the highest ACE index; however, the OPT+Mg treatment still demonstrated a substantial improvement over the FP treatment, indicating that Mg plays a role in maintaining elevated levels of bacterial diversity. During the third growth season (S3), both OPT and OPT+Mg displayed comparable but higher ACE index values than FP. This finding indicates the consistent positive impact of optimized nutrient management and Mg supplementation on microbial diversity. Similarly, for the Shannon index (Fig. 3D), the microbial diversity in leaf samples varied significantly (p < 0.05) across different treatments and growth stages. In stage S1, the Shannon index for all treatments (FP, OPT, OPT+Mg) was consistent. During S2, OPT and OPT+Mg exhibited higher microbial diversity; in S3, FP had the lowest diversity. In general, the optimized treatments (OPT and OPT+Mg) tended to support higher microbial diversity than the FP treatment, highlighting the positive effects of optimizing agricultural practices on the phyllosphere microbiome.

### 3.3 Effect of nutrient management on phyllosphere bacterial community composition

**Fig. 4:**
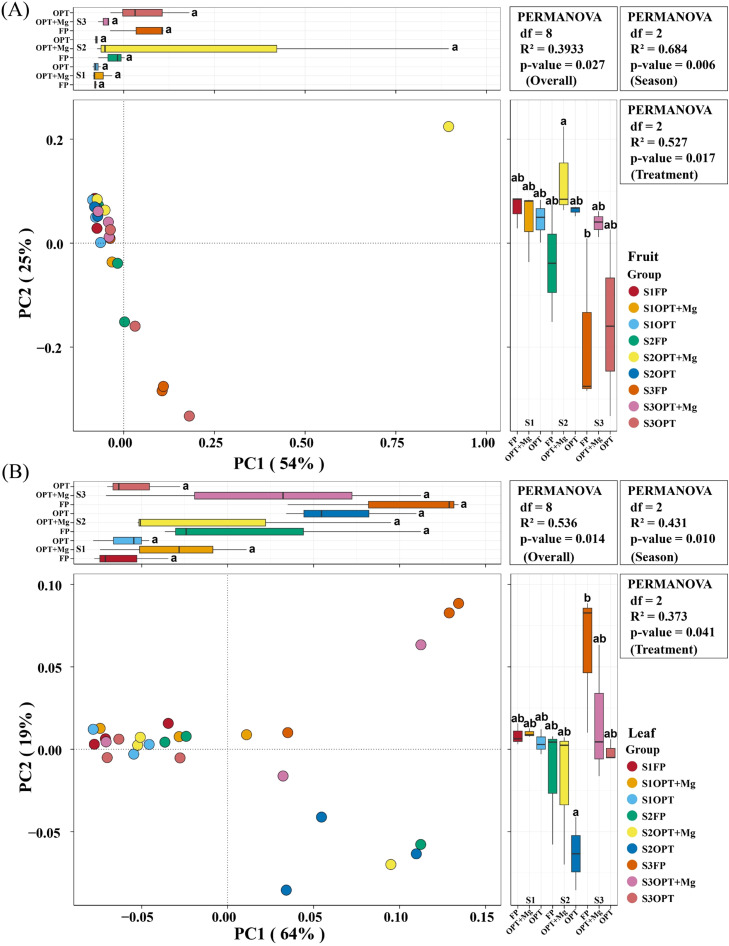
Changes in phyllosphere bacterial community structures and composition under different treatments across various growth stages; (A) fruit sample; (B) leaf sample. Here S1, S2, S3 represent various growth stages (seasons) in June, July, September, respectively. While, FP, OPT, and OPT+Mg represents different treatments.

We used Principal Coordinate Analysis (PCoA) to measure phyllosphere bacterial community similarity in leaf and fruit samples across treatments and growing seasons (Fig. 4). Fruit samples displayed shifts in ordination space influenced by both treatments and developmental stage, although clustering was not fully discrete, indicating significant differences were observed among the different treatments growing seasons. The first two principal coordinates, i.e., PC1 and PC2 explained 54 % and 25 % pf the total variation in bacterial composition, respectively. We analyzed microbial community structure using the PCoA plot with PERMANOVA results (Permutational Multivariate Analysis of Variance; R² = 0.393, p = 0.027) highlighting statistically significant differences for bacterial community when comparing different groups (Fig. 4A).

Likewise, the PCoA plot clearly separated the bacterial communities for leaf samples, representing substantial differences among the different treatments and growth stages. The first principal coordinate (PC1) accounted for 64 % of the variation, whereas the second principal coordinate (PC2) contributed 19 % of the variation recorded in bacterial composition. PERMANOVA results (R² = 0.536, p = 0.014) also confirmed that treatment and season significantly affected the phyllosphere bacterial community structure in leaf samples. Hence, the PCoA plots and PERMANOVA analyses revealed that treatment and season significantly influence the microbial community structures in fruit and leaf samples. This underscores the effectiveness of the different nutrient management practices in enhancing microbial diversity and altering community structure in both fruits and leaves (Fig. 4B).

### 3.4 Variability of phyllosphere bacterial community

**Fig. 5:**
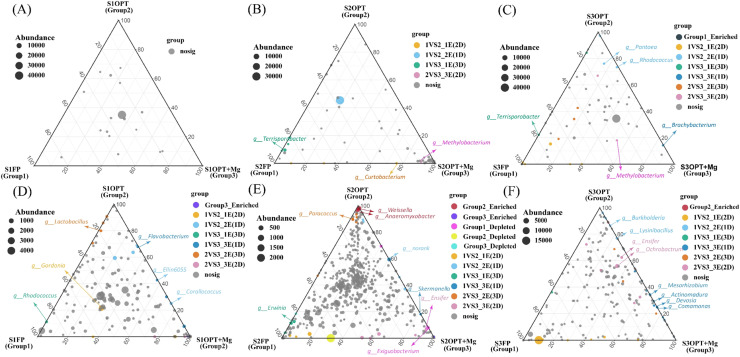
Ternary plot representing the phyllosphere bacterial communities with significant differences in relative abundance under different treatments across various growth stages; (A-C) fruit sample; (D-F) leaf sample. Here S1, S2, S3 represent various growth stages in June, July, September, respectively. While, FP, OPT, and OPT+Mg represents different treatments.

Ternary plot analysis was performed to identify the specific enriched and depleted bacterial genera under different treatments. The ternary plot analysis for fruit samples (Fig. 5A-C) revealed significant changes in bacterial community composition under various treatment conditions. We did not observe any significant enriched or depleted genera for S1 (Fig. 5A). In contrast, S2 fruit samples were enriched with Curtobacterium under FP treatment, while OPT and OPT+Mg were enriched with Terrisporobacter and Methylobacterium, respectively (Fig. 5B). For S3, fruit samples showed a notable shift in phyllosphere bacterial community, with FP enriched with Terrisporobacter, OPT enriched with Pantoea and Rhodococcus, and OPT+Mg treatment enriched the abundance of Brachybacterium and Methylobacterium (Fig. 5C). These findings highlight the different effects of reduced N.P.K input (OPT) and magnesium supplementation (OPT+Mg) on the bacterial composition of fruit samples.

Ternary plot analysis of leaf samples showed that different treatments had specific phyllosphere bacterial community compositions (Fig. 5D-F). In S1, FP was enriched with Rhodococcus and Gordonia, indicating these genera thrive under this condition. OPT favored Corallococcus and Lactobacillus, while OPT+Mg enhanced Flavobacteria and Ellin6055, suggesting nutrient management positively influences these bacteria (Fig. 5D). For S2, FP was enriched with Erwinia but showed depletion of Exiguobacterium. OPT treatment promoted a diverse community, including Norank, Paracoccus, Weissella, and Anaeromyxobacter, whereas OPT+Mg significantly enriched Skermanella and Ensifer (Fig. 5E). In S3 leaf samples, FP supported Candidatus, OPT enriched Burkholderia and Lysinibacillus, and OPT+Mg boosted Actinomadura, Comamonas, Devosia, Mesorhizobium, Ochrobactrum, and Ensifer (Fig. 5F). These findings highlight the differential effects of optimized treatments and magnesium addition on bacterial genera, promoting distinct microbial communities in leaf samples.

### 3.5 The co-occurrence network of the phyllosphere bacterial community

**Fig. 6:**
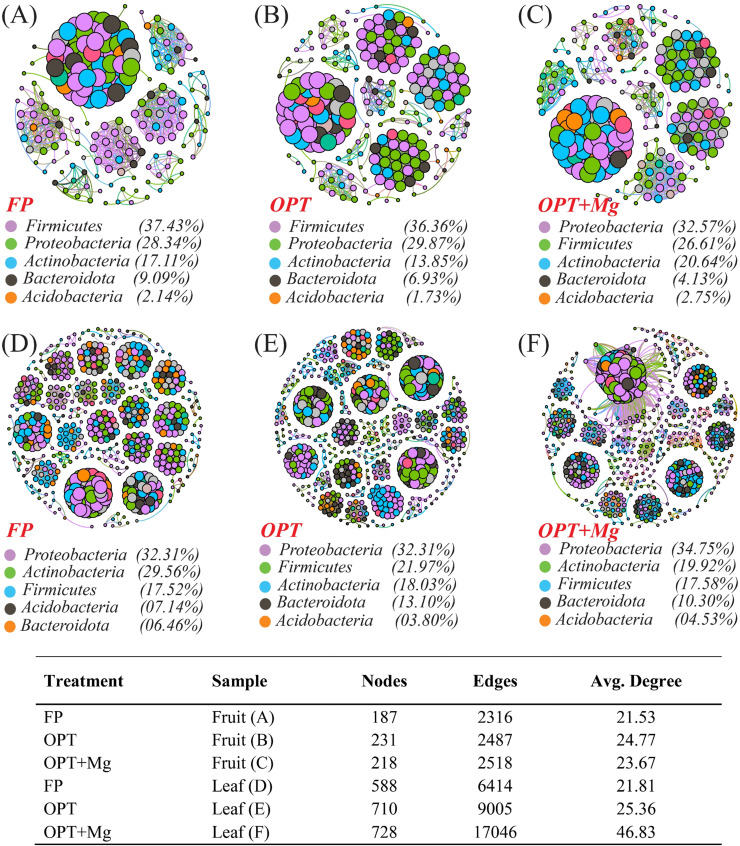
Co-occurrence network of phyllosphere bacterial communities based on correlation analysis for different treatments; (A-C) fruit sample; (D-F) leaf sample. The nodes in the network are colored based on phylum, and size of each node is proportional to relative abundance of specific taxa. Here S1, S2, S3 represent various growth stages in June, July, September, respectively. While, FP, OPT, and OPT+Mg represents different treatments.

The bacterial community structure and network topological features in the fruit samples varied across the different treatments. For example, FP displayed a network with 187 nodes, 2316 edges, and an average degree of 21.532, dominated by Firmicutes (37.43 %), Proteobacteria (28.34 %), and Actinobacteria (17.11 %) (Fig. 6A). By contrast, OPT had a slightly more extensive network with 231 nodes and 2487 edges, and a higher average degree of 24.77, with Firmicutes (36.36 %), Proteobacteria (29.87 %), and Actinobacteria (13.85 %) being the most prevalent (Fig. 6B). In OPT + Mg, the network comprised 218 nodes, 2518 edges, and an average degree of 23.679, with a notable increase in Proteobacteria (32.57 %) and Actinobacteria (20.64 %). Compared with OPT, the OPT+Mg treatment had more edges and a higher average degree, showing greater network connectivity within the optimized regime. Because co-occurrence networks are based on correlations, these results describe patterns rather than prove cause–effect relationships (Fig. 6C).

For the leaf samples, the network characteristics were markedly more complex than in the fruit samples. FP exhibited a network with 588 nodes, 6414 edges, and an average degree of 21.816, with Proteobacteria (32.31 %), Actinobacteria (21.94 %), and Firmicutes (17.52 %) being predominant (Fig. 6D). OPT showed a substantial increase in network size with 710 nodes and 9005 edges, and an average degree of 25.366, reflecting a diverse microbial community dominated by Proteobacteria (32.31 %) and Actinobacteria (21.71 %) (Fig. 6E). OPT + Mg presented the most complex network with 728 nodes, 17,046 edges, and an average degree of 46.83, indicating a higher microbial interactions, especially among Proteobacteria (34.75 %) and Actinobacteria (19.42 %) (Fig. 6F). The addition of Mg in the OPT increased network connectivity and complexity, highlighting its impact on promoting robust microbial communities in leaf samples. Since co-occurrence networks are correlation-based, these findings describe community patterns but do not confirm direct causal effects.

### 3.6 Functional analysis

**Fig. 7:**
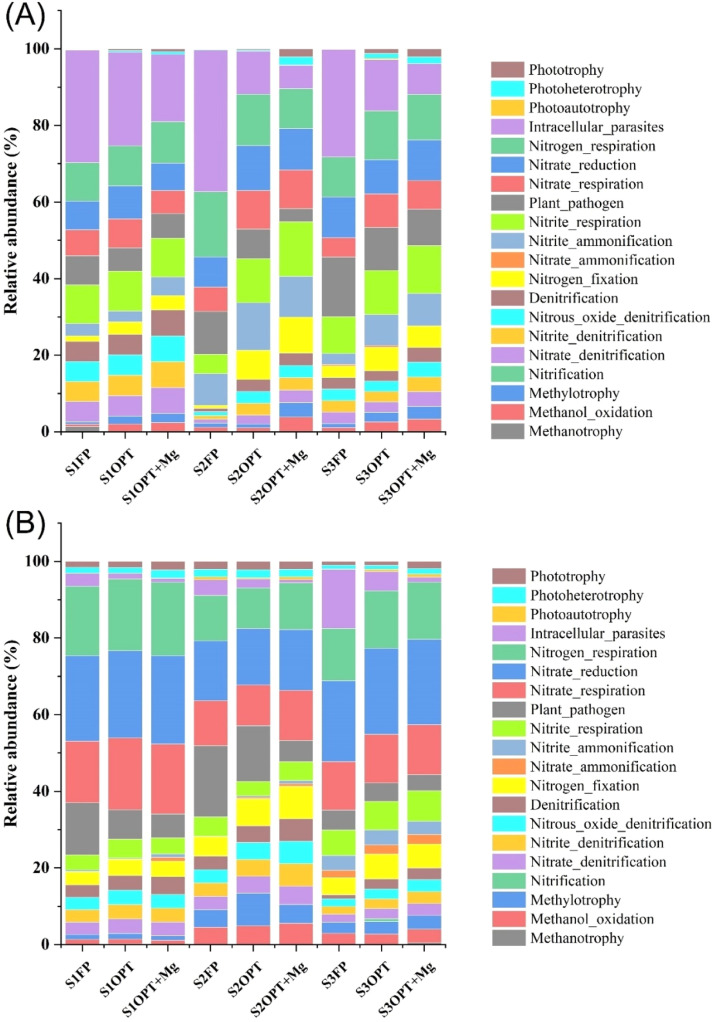
Functional Annotation of Prokaryotic Taxa (FAPROTAX) analysis to evaluate the relative abundance of phyllosphere endophytic bacterial community; (A) fruit sample; (B) leaf sample.

We used FAPROTAX to predict functional profiles of the phyllosphere bacterial community (Fig. 7). The major predicted functions were primarily related to carbon (C) and nitrogen (N) cycling, along with the presence of functional groups associated with plant pathogens and intracellular parasites. We found that predicted relative abundances of phyllosphere bacterial communities involved in C-cycle and N-cycle were higher under OPT and OPT+Mg than under FP. For example, higher predicted relative abundance of phyllosphere bacterial communities controlling N-fixation in both fruit and leaf samples. In contrast, taxa annotated as plant pathogens and intracellular parasites had lower predicted relative abundances under OPT+Mg than under OPT. Additionally, the intracellular parasites showed a reduction with Mg addition across all samples, suggesting that Mg may help mitigate intracellular parasitic bacterial activity. These results highlight the differential impacts of Mg on various functional groups within the phyllosphere bacterial community, emphasizing its potential benefits in enhancing nitrogen fixation and reducing pathogens and parasites.

## 4 Discussion

Excessive and unbalanced use of N.P.K fertilizers has been shown to negatively impact plant-associated microbial communities, including those in the phyllosphere, by altering nutrient stoichiometry and disrupting plant–microbe interactions (Trivedi et al., 2020). Such practices can reduce microbial diversity and favor the proliferation of opportunistic or pathogenic taxa. Therefore, optimizing nutrient management is essential for supporting the balanced and functional phyllosphere microbiome, which contributes to plant growth, stress resilience, and activation of defense pathways against pathogens (Remus‐Emsermann and Schlechter, 2018; Stone et al., 2018).

We found contrasting effects of nutrient management on the phyllosphere bacterial community, particularly in terms of diversity in leaf and fruit samples. The relative abundances of key phyllosphere bacterial taxa, especially Actinobacteriota, Bacteroidota, and Chloroflexi were significantly increased under OPT, and especially under OPT+Mg treatment (Fig. 2). These findings are consistent with previous studies showing that nutrient management is an important factor driving phyllosphere microbiome assembly, as balanced inputs reduce the risk of nutrient excess or imbalance and thereby promote more stable microbial enrichment (Li et al., 2022; Zhu et al., 2022). Besides, Mg availability may increase the uptake of essential nutrients, such as N and P, for the phyllosphere bacterial community (Li et al., 2022; Wang et al., 2020). Therefore, this balanced nutrient supply benefits the growth and proliferation of a broader range of bacterial taxa in the phyllosphere, leading to an increased relative abundance of the bacterial community.

It is interesting to note that nutrient management, especially OPT and OPT+Mg led to an increased relative abundance of beneficial phyllosphere bacterial communities. The current study showed that the relative abundances of key phyllosphere bacterial communities, such as Actinobacteriota, Bacteroidota, and Chloroflexi, significantly increased under OPT and OPT+Mg treatments. Specifically, Actinobacteria under OPT+Mg, and this shift may be attributed to Mg supplementation, which enhances nutrient availability and creates a favorable environment for microbes involved in organic matter decomposition and nutrient cycling (Raimi et al., 2023; Shi et al., 2023). Bacteroidota also play a vital role in processing complex organic matter substances, such as plant-derived polysaccharides (Ji et al., 2023; Li et al., 2023b). Chloroflexi can degrade recalcitrant organic compounds and contribute to soil carbon or nitrogen cycling (Li et al., 2023b; Raimi et al., 2023). Moreover, OPT+Mg treatment exhibited the most overlap and the highest number of unique OTUs compared to other treatments, indicating its efficiency in sustaining a diverse microbial community.

The alpha diversity indices also revealed a more diverse bacterial community under OPT and OPT+Mg compared to FP (Fig. 3), particularly during the S2 stage, when OPT+Mg produced the highest richness and diversity values in fruit samples. By contrast, at S3 the Shannon index was numerically higher in FP, although not significantly different from OPT or OPT+Mg. This stage-specific pattern indicates that improved agricultural management beneficially impacts phyllosphere microbial population, with the strongest effects occurring during mid-development. Nutrient management practices usually result in precise and judicious fertilizer use, which may result in a favorable microbial community environment. It has been documented that nutrient supplementation can improve microbial diversity by supplying the necessary nutrients for the growth and metabolism of the microbial population (Abdel-Aziz et al., 2016). For instance, the researchers (Leff and Fierer, 2013) confirmed that nutrient amendments significantly increase the phyllosphere bacterial diversity.

Mg contributes to microbial diversity through multiple physiological roles. Beyond its well-established function in chlorophyll production and enzyme activation, Mg regulates carbon source–sink dynamics by facilitating phloem loading and sucrose transport from leaves to fruits (Cakmak and Kirkby, 2008; Hermans et al., 2006). Enhanced carbohydrate translocation increases soluble sugar pools in fruit tissues, which expand ecological niches and provide substrates that support a broader range of microbial taxa. This mechanism likely underlies the marked increase in fruit microbial diversity under OPT+Mg during the S2 stage, when carbon allocation demand peaks. Mg also modulates plant immune responses, including the salicylic acid (SA) signaling pathway (Ahmed et al., 2023; Guo et al., 2016). Adequate Mg nutrition promotes SA biosynthesis and defense gene expression, strengthening systemic acquired resistance. This immune priming may suppress opportunistic pathogens while favoring beneficial commensals and endophytes, thereby contributing to the more balanced phyllosphere microbiome observed under OPT+Mg treatment. Previous findings have confirmed that Mg addition contributes to vigorous plant growth, encouraging diverse microbial communities (Yang et al., 2023). Hence, the integrated use of Mg with OPT (OPT+Mg) may improve plant growth and result in a higher bacterial community diversity in the leaf and fruit samples of the current study.

Generally, these optimized nutrient management practices often aim to minimize chemical fertilizers that reduce microbial abundance and diversity (Ottesen et al., 2009). Various studies have shown the benefits of reduced chemical input farming in improving the phyllosphere microbial diversity. For instance, Perazzolli et al. (2014) found that grapevine leaves harbor higher microbial diversity under reduced pesticide input than conventional practices (Perazzolli et al., 2014). Moreover, we observed a sharp increase in microbial diversity in fruit samples under OPT+Mg during the S2 stage, which then declined slightly at S3 but remained higher than S1. Recent work on pomelo fruit biochemistry (Su et al., 2024) has shown that as fruits progress through these stages, pulp accumulates phenolics and mineral bioavailability while phytic acid levels decline, and these effects are further enhanced under Mg-supplemented fertilization. Such developmental and nutrient-driven shifts expand ecological niches and resource pools within the fruit, particularly through increased carbohydrate translocation and antioxidant accumulation, thereby favoring the recruitment and persistence of a more diverse endophytic microbiome. Nevertheless, optimized treatments result in improved microhabitat conditions (i.e., humidity, nutrient supply) on leaf and fruit surfaces, which are important for microbial colonization and diversity (Vorholt, 2012). Researchers also suggested that plants under optimal growth conditions support a more diverse microbial population in the phyllosphere due to improved microhabitat conditions (Rastogi et al., 2013). Plants grown under optimal management conditions experience less stress from nutrient constraints, leading to healthy and more stable phyllosphere environments. Reduced plant stress may positively impact microbial diversity (Lindow and Brandl, 2003). Hence, our current results align with previous findings that stressed plants (due to suboptimal growing conditions) had lower microbial diversity than those under optimized management (Copeland et al., 2015). In summary, the increased phyllosphere microbial diversity observed with optimized treatments (OPT and OPT+Mg) in our study is consistent with existing literature that highlights the benefits of balanced nutrient management, reduced chemical inputs, and improved microhabitat conditions on microbial diversity. These findings highlight that Mg contributes not only to plant vigor but also to microbiome assembly through its roles in carbon partitioning and immune regulation, reinforcing the importance of sustainable nutrient management for promoting healthy and diverse microbial communities.

The PCoA plot based on fruit and leaf samples showed a clear separation of microbial communities among the different treatments (Fig. 4). The clustering of bacterial communities under different treatments indicated a strong response to nutrient availability. In high-nutrient environments (e.g., FP), a few fast-growing microbial taxa dominate and reduce overall microbial diversity (Leff and Fierer, 2013). Conversely, decreased nutrient input (such as the OPT and OPT+Mg treatments in the current study) can promote a diverse microbial community by reducing competitive exclusion, thereby maintaining a wider range of functional groups and species coexistence (Tilman, 1982). Such niche differentiation leads to improved microbial diversity under lower nutrient conditions. Additionally, Mg addition further enhances the microbial community indirectly by improving plant health and exudate composition, which may favor a large number of microbial populations (Hooper and Vitousek, 1997).

A ternary plot analysis was conducted to determine the specific bacterial genera that were enriched or depleted under different nutrient management treatments for the phyllosphere endophytic bacterial community (Fig. 5). We found that nutrient management (OPT and OPT+Mg) had a positive impact on and regulated the beneficial phyllosphere endophytes in leaf and fruit samples. For fruit samples, under the FP treatment, Curtobacterium and Terrisporobacter were enriched. Curtobacterium species are known as pathogens that cause diseases of blight and bacterial wilt in different crops (Puia et al., 2021). Likewise, Terrisporobacter is also reported as a pathogenic bacterium (Mitchell et al., 2023) that reflects the negative impacts of FP treatment on the bacterial community. These results align with previous studies indicating that excessive nitrogen fertilization can disrupt phyllosphere microbial communities, leading to an increased abundance of pathogenic bacteria (Leff et al., 2015). Such practices often lead to an environment where pathogenic microorganisms may invade and cause different plant diseases (Friesen, 2013). In contrast, OPT treatment resulted in beneficial genera, such as Pantoea and Rhodococcus. The Pantoea species act as a biocontrol agent and produces metabolites that enhance the auxin activity and promote plant growth, as found in the isolates of Pantoea agglomerans and Pantoea ananatis (Kim et al., 2012; Luziatelli et al., 2020). Likewise, Rhodococcus species produce cytokinins and strigolactones that improve plant growth and increase resistance to stress‑induced bacterial infections (Kuhl et al., 2021). Under OPT+Mg treatment, Methylobacterium and Brachybacterium species were enriched which have important functions in carbon cycling and growth promotion (Borzyskowski et al., 2018; Gontia et al., 2011). These species help in root colonization and improve plant nutrient uptake (Kim et al., 2010). Brachybacterium species also have beneficial interactions with host plants, improving nutrient acquisition, stress resistance, and plant growth (Alexander et al., 2021). For example, Brachybacterium saurashtrense has essential implications for the physiological and biochemical activities of peanut plants under N-starvation, indicating its importance in plant development and yield production (Alexander et al., 2021). These phyllosphere Brachybacterium species boost plant growth and stress resistance, consistent with our observations of beneficial bacterial communities in the phyllosphere under optimized nutrient management (Gupta and Patil, 2020).

For leaf samples, FP treatment enriched genera such as Gordonia, Rhodococcus, Erwinia, and Candidatus. Gordonia species, although occasionally opportunistic pathogens, pose a risk for infections, and their presence necessitates careful monitoring (Drzyzga, 2012). Rhodococcus, known to cause leafy gall disease, can lead to significant economic losses due to abnormal plant tissue proliferation (Goethals et al., 2001). Erwinia species are well-known phytopathogens, underscoring the importance of effective nutrient management to mitigate these risks (Pérombelon, 2002). The presence of Candidatus species, often associated with severe plant diseases like Huanglongbing in citrus, indicates a potential threat to plant health under FP conditions (Bové, 2006). In contrast, OPT treatment resulted in the enrichment of various beneficial genera, e.g., Corallococcus, Lactobacillus, Paracoccus, and Weissella. Corallococcus species suppress pathogenic fungi and improve plant growth (Li et al., 2017). Lactobacillus plantarum and other lactobacilli facilitate the control of pathogenic microbes and enhance plant health under OPT treatment (Daranas et al., 2019). The Paracoccus species helps in nutrient cycling and improving plant growth (Sahoo et al., 2019). In contrast, the Weissella species have probiotic potential, leading to plant resilience against stresses (Fusco et al., 2015). Under OPT+Mg treatment, Flavobacteria, Ensifer, and Actinomadura species were significantly abundant. Flavobacteria species are involved in plant growth promotion, enhanced drought resistance, and nutrient cycling (Kolton et al., 2016), thus providing several benefits to plants under OPT+Mg treatment. The Ensifer species regulate N-fixation, which results in increased N-availability and stimulates plant growth (Rogel et al., 2001). The Actinomadura species also play various functions, such as nutrient cycling, and release useful compounds that improve soil health and plant growth (Bhatti et al., 2017).

We found that OPT and OPT+Mg improved the network connectivity and complexity of the bacterial community compared to conventional FP practice (Fig. 6). This suggests that these nutrient managements favors a more interconnected and interactive bacterial community. This agrees with earlier studies, which have shown that higher microbial diversity and interactions can be sustained by balanced nutrient inputs since this ensures a steady inflow of essential nutrients (Finkel et al., 2017; Leff et al., 2015). In contrast, conventional high N.P.K input (FP) may result in nutrient imbalances or excesses that could suppress microbial growth and/or alter community composition (Hartman et al., 2017). Mineral fertilizers at high levels, especially nitrogen (N), reduce the overall diversity of bacterial communities (Zeng et al., 2016). This lower diversity of the microbiome results in a more simplified network of microbial interactions. High-N.P.K inputs lead to changes in soil nutrient availability, which may substantially affect the composition and abundance of bacterial taxa (Pan et al., 2014). Mg is essential for several cellular functions, including enzyme activation, ribosome stabilization, and nucleic acid synthesis. Its presence in the OPT+Mg treatment probably increased metabolic activities and interactions among the bacterial community, stabilizing more complex and resilient networks (Romero et al., 2014). Previous studies have demonstrated the influence of Mg on microbial communities by promoting microbial growth and activity (Yang et al., 2023), leading to more complex network among microorganisms. The higher degrees of network connectivity and complexity in OPT, as well as in OPT+Mg treatments, indicated a more functionally diverse phyllosphere bacterial community. These communities are known to enhance plant health through growth promotion, stress tolerance, and pathogen protection (Berg et al., 2014; Vorholt, 2012). Increased microbial interactions could translate into enhanced nutrient cycling and increased plant-microbe synergy, thereby supporting sustainable agriculture and ecosystem health.

The current study also showed that Mg addition consistently increased the relative abundance of phyllosphere bacterial population controlling C- and N-cycling (for instance, N-fixation) and suppressed parasites and pathogens (Fig. 7). The increase in relative abundance of bacterial community associated with N-fixation is caused by Mg’s contribution to chlorophyll production that is crucial for photosynthesis and energy provision for N-fixing bacteria (Mengel and Kirkby, 1987). Moreover, Mg plays a key role in numerous enzymes involved in N-fixation and regulates vigorous root development and nodule formation, thus facilitating the symbiotic relationship with N-fixing bacteria (Abreu et al., 2016; Cakmak and Yazici, 2010). Mg also aids in plant immunity by the synthesis of defense compounds and strengthens the cell walls, making it harder for plant pathogens to invade (Fujikawa et al., 2021). Furthermore, Mg improves uptake efficiency of essential nutrients, boosting the overall plant health and resistance to diseases (Marschner, 2012). By improving plant stress tolerance and antioxidant production, Mg mitigates the impact of destructive microbiota, ensuring healthier and more productive crops.

## 5 Conclusions

The current study conclusively demonstrates that long-term optimized nutrient management, particularly Mg addition, can enhance the diversity, community composition, and predicted functional potential of phyllosphere endophytic bacterial communities in pomelo orchards. Treatments with OPT and especially OPT+Mg supported a richer and more interactive microbial community compared to FP. The observed enrichment of potentially beneficial microbial taxa and metabolic functions underscores the role of balanced nutrient input in promoting a diverse phyllosphere microbiome. While our findings are limited to microbial parameters, they suggest that Mg-inclusive fertilization practices may contribute to a healthier aboveground microbial environment. Future research should investigate how these microbial changes influence plant health, disease resistance, and ecosystem processes, including nutrient cycling and productivity, across broader temporal and spatial scales.

While this long‑term field experiment provides valuable insights into how optimized nutrient management and Mg supplementation influence the phyllosphere microbiome of pomelo, several limitations should be acknowledged. First, despite the randomized block design, other unmeasured factors, such as microclimatic variation, soil heterogeneity, and tree age or health, may confound the observed microbial responses. Future studies should incorporate multi‑site experiments, additional environmental measurements and larger sample sizes to better disentangle the effects of nutrient management from other abiotic and biotic drivers. Second, the functional profiles inferred from FAPROTAX and the co‑occurrence network analyses are predictive and correlation‑based; they do not directly measure metabolic activity or causal relationships. Moreover, to ensure adequate statistical power in the co-occurrence analysis, samples were pooled across seasons, which may obscure time-specific microbial interactions or seasonality effects. These findings should therefore be interpreted as reflecting persistent or core associations, while finer temporal dynamics remain to be resolved. Therefore, these findings should be interpreted as hypotheses that require verification through controlled greenhouse or microcosm experiments, metagenomic or metatranscriptomic analyses, and targeted ecological assays. Recognizing these limitations will help guide subsequent research toward a more comprehensive understanding of nutrient–microbiome interactions and their implications for pomelo orchard management.

## Data availability

All data generated or analyzed during this study are included in this article. The raw reads of sequencing data are available at NCBI BioProject SRA database under the accession number PRJNA1147855.

## Funding

This research was supported by the Industrial Parks from the Ministry of Agriculture and Rural Affairs of China for the "Construction of the Modern Agricultural Industrial Park in Pinghe County, Fujian Province" (FJPH2021–28), the National Citrus Production System in China (CARS-26–01A), the Department of Science and Technology of Fujian Province (2021N0008), and Open Research Foundation of International Magnesium Institute (IMI2018–09).

## CRediT authorship contribution statement

**Muhammad Atif Muneer:** Writing – original draft, Investigation, Conceptualization. **Rong Huang:** Writing – review & editing, Methodology. **Yan Xiaojun:** Writing – review & editing. **Ziqin Pang:** Writing – review & editing, Methodology, Data curation. **Muhammad Zeeshan Munir:** Writing – review & editing. **Baoming Ji:** Writing – review & editing, Conceptualization. **Liangquan Wu:** Writing – review & editing, Data curation. **Chaoyuan Zheng:** Writing – review & editing, Conceptualization, Supervision.

## Declaration of competing interest

The authors declare that they have no known competing financial interests or personal relationships that could have appeared to influence the work reported in this paper.

## Data Availability

Data will be made available on request.
